# Endometrial regenerative cells with galectin-9 high-expression attenuate experimental autoimmune hepatitis

**DOI:** 10.1186/s13287-021-02604-2

**Published:** 2021-10-15

**Authors:** Hongda Wang, Yiming Zhao, Bingbing Ren, Yafei Qin, Guangming Li, Dejun Kong, Hong Qin, Jingpeng Hao, Daqing Sun, Hao Wang

**Affiliations:** 1 Department of General Surgery, Tianjin Medical University General Hospital, 154 Anshan Road, Heping District, Tianjin, 300052 China; 2 Tianjin General Surgery Institute, Tianjin, China; 3 Department of Pediatric Surgery, Tianjin Medical University General Hospital, 154 Anshan Road, Heping District, Tianjin, 300052 China; 4 Department of Anorectal Surgery, The Second Hospital of Tianjin Medical University, Tianjin, China; 5 Department of Hepatobiliary Surgery, Union Hospital, Tongji Medical College, Huazhong University of Science and Technology, Wuhan, China

**Keywords:** Endometrial regenerative cell, Concanavalin A-induced hepatitis, Galectin-9, Autoimmune hepatitis

## Abstract

**Background:**

Autoimmune hepatitis (AIH) is a T cell-mediated immune disease that activates abnormally against hepatic antigens. We have previously reported that endometrial regenerative cells (ERCs) were a novel source of adult stem cells, which exhibiting with powerful immunomodulatory effects. Galectin-9 (Gal-9) is expressed in ERCs and plays an important role in regulating T cell response. This study aims to explore the role of ERCs in attenuation of AIH and to determine the potential mechanism of Gal-9 in ERC-mediated immune regulation.

**Methods:**

ERCs were obtained from menstrual blood of healthy female volunteers. In vitro, ERCs were transfected with lentivirus vectors carrying LGALS9 gene and encoding green fluoresce protein (GFP-Gal-9-LVs) at a MOI 50, Gal-9 expression in ERCs was detected by ELISA and Q-PCR. CD4^+^ T cells isolated from C57BL/6 mouse spleen were co-cultured with ERCs. The proliferation of CD4^+^ T cells was detected by CCK-8 kit and the level of Lck/zap-70/LAT protein was measured by western blot. Furthermore, AIH was induced by ConA in C57BL/6 mice which were randomly assigned to untreated, unmodified ERC-treated and Gal-9 high-expressing ERC-treated groups. Histopathological score, liver function, CD4^+^/CD8^+^ cell infiltration in liver tissues, the proportion of immune cells in the spleen and liver, and ERC tracking were performed accordingly to assess the progression degree of AIH.

**Results:**

After transfecting with GFP-Gal-9-LVs, Gal-9 expression in ERCs was significantly increased. Additionally, Gal-9 high-expressing ERCs effectively inhibited CD4^+^ T cell proliferation and downregulated CD4^+^ T cell active related proteins p-Lck/p-ZAP70/p-LAT in vitro. Furthermore, treatment with Gal-9 high-expressing ERCs restored liver function, ameliorated liver pathological damage, inhibit CD4^+^ and CD8^+^ T cell proliferation and suppress Th1 and Th17 cell response in the hepatitis mice. In addition, Gal-9 high-expressing ERCs further markedly enhanced the level of IL-10 but reduced the levels of IFN-γ, TNF-α, and IL-4 in mouse sera and liver. Cell tracking also showed that ERCs could migrate to the damaged liver organs.

**Conclusions:**

The results suggested that Gal-9 was an essential modulator, which was required by ERCs in regulating T cell response and attenuating ConA-induced experimental hepatitis. And also, it provides a novel idea for the clinical treatment of AIH.

## Introduction

Autoimmune hepatitis (AIH) is a chronic progressive hepatic disease mediated by autoimmune response [1]. It is reported that the point prevalence up to be 10–25 per 100,000 population in the European region, and 5–25 per 100,000 population in the Asia–Pacific region [2], and the specific pathogenesis of AIH is still unclear. The conceptual framework of AIH postulates an environmental agent that triggers a cascade of T-cell-mediated events directed at liver cells, and persistent inflammation within the liver can result in scaring, eventually leading to liver cirrhosis.

At present, the main therapeutic drugs are glucocorticoids and azathioprine, which are mainly used for induction of remission or for maintenance [3]. But patients generally relapse after cessation of the therapy, and also face the inevitable side effects of steroids [4, 5]. Studies have also shown that although AIH patients initially respond well to immunosuppression, their life expectancy is much lower than that of the general population [5, 6]. Thus, it is worthwhile exploring new therapies for the attenuation of AIH.

Mesenchymal stromal cells (MSCs) are one kind of adult stem cells, and with the abilities of multiple-differentiation, directional migration, and immune-regulation [7]. In recent years, a number of studies have applied MSCs to the experimental treatment of AIH and have achieved certain effects [8, 9]. However, the current application of MSCs still has some limitations. For example, the acquisition method for MSCs is mostly invasive, and it is difficult to obtain MSCs in large quantities at one time [10, 11].

Endometrial regenerative cells (ERCs) are obtained from menstrual blood and regarded as a novel source of adult stem cells [12]. Compared with traditional MSCs, ERCs are resource-rich, easy and non-invasive to obtain, and highly expandable in vitro, while retaining the immunoregulatory function [13]. Clinical studies have shown that the administration of ERCs is relatively safe. And no side effects have been reported related to ERC treatment [14]. Previous studies performed by our research group and others have also shown that ERCs have therapeutic effects in a variety of disease models, such as cardiac transplantation [15], renal ischemia–reperfusion injury [16], and inflammatory bowel disease [17], etc. However, their therapeutic effects and regulatory mechanisms in AIH are still unclear.

Galectin-9 (Gal-9) is a 36 kDa β-galactoside lectin protein. Gal-9 is an important member of the Galectin family and a natural ligand for immune checkpoint T-cell immunoglobulin and mucin-domain containing-3 (TIM-3) [18]. Gal-9 has been proven to play a therapeutic role in a variety of autoimmune disease models and transplant rejection models [19–21]. It mainly achieves its effects by inhibiting the proliferation of CD4^+^ Th1, CD4^+^ Th17 cells and promoting the population of Foxp3^+^ T regulatory cells (Tregs) [22]. Based on the preliminary experiments of our research group, we have verified the expression of Gal-9 protein on ERCs. Consistently, the main pathogenesis of AIH is the abnormal activation of CD4^+^ T cells against self-antigens and the absence of Tregs [1]. In view of the existing literature, we have raised the question of whether ERCs can effectively attenuate AIH, and whether the therapeutic effect of ERCs is mainly achieved by Gal-9 expression.

Overall, the purpose of our study is to evaluate the therapeutic effect of ERCs on experimental AIH and to explore the potential mechanisms underlined, thus providing a novel insight for the clinical treatment of AIH.

## Materials and methods

### Isolation and phenotype identification of ERCs

The extraction and separation of ERCs followed the protocol described previously [23]. In short, menstrual blood from female volunteers (20–40 years old) was collected on the first day of the menstrual cycle. The mononuclear cell suspension isolated from menstrual blood was added to a complete DMEM medium containing 1% penicillin/streptomycin (Solarbio, China) and 10% Fetal Bovine Serum (Corning, Australia), and then inoculated into a 6-well plate. Then, the plate was placed in a constant temperature incubator (37 °C and 5% CO2), and the cells adhere to the plate after 24 h. We took photos of ERCs from different generations and collected the fifth-generation ERCs to detect their surface markers (CD29, CD44, CD45, CD31, and CD90). All ERCs transplanted into mice are from the same fifth-generation of ERCs.

### Animals

6–8 Week male C57BL/6 mice weighing 22–25 g were purchased from the China Food and Drug Inspection Institute (Beijing, China). All animal research and experimental procedures were carried out following the guidelines of the China Association for the Protection of Animals and the protocol approved by the Animal Care and Use Committee of Tianjin Medical University (Tianjin, China). All animals were kept in the constant temperature room of Tianjin Institute of General Surgery, where they can freely obtain food and drinking water.

### ERCs transfected with GFP-Gal-9-LVs

Lentivirus vector carrying the LGALS9 gene and encoding green fluoresce protein (GFP-Gal-9-LVs) was constructed by GeneChem Inc, Shanghai, China Gene. Second-generation of ERCs (4 × 10^5^/ml) were harvested, planked and transfected with GFP-Gal-9-LV (MOI = 50) for 12 h. And then, the culture medium was washed away and the transfected ERCs were cultured in complete medium for additional 60 h. After 72 h, these transfected ERCs were observed with a fluorescence microscope to evaluate the transfection efficiency, and the successful transfected ERCs were further cultured in a complete medium, which containing 2 mg/ml puromycin, to purify the transfected cells.

Then, the ERCs transfected with GFP-Gal-9-LVs and screened with puromycin were collected. Gal-9 protein and mRNA expression in ERCs were detected by ELISA and RT-PCR.

### Real-time polymerase chain reaction (RT-PCR)

All the experimental protocols were carried out based on the manufacturer's instructions. Firstly, ERCs and GFP-Gal-9-LVs transfected ERCs were collected. The total RNA in the above ERCs was extracted (DP430 TIANGEN BIOTECH, Beijing, China), and then the purity and concentration of RNA were evaluated at 230, 260, and 280 nm by an ultraviolet spectrophotometer. The extracted RNA was reversely transcribed into cDNA by a FastKing gDNA Dispelling RT SuperMix (TIANGEN BIOTECH, Beijing, China). RT-PCR was used to evaluate the expression of the Gal-9 gene with Real Universal Color PreMix (SYBR Green) kit (TIANGEN BIOTECH, Beijing, China). The relative expression of the Gal-9 gene was analyzed by the 2^−ΔΔCT^ method. Primer sequence:

$$\begin{array}{*{20}l} \text{Gal-}9 & \begin{gathered} {\mathrm{Forward}}\;{\mathrm{primer:}}\;{\mathrm{GGACGGACTTCAGATCACTGT;}} \hfill \\ {\mathrm{Reverse}}\;{\mathrm{primer:}}\;{\mathrm{CCATCTTCAAACCGAGGGTTG;}} \hfill \\ \end{gathered} \\ {{\mathrm{GAPDH}}} & \begin{gathered} {\mathrm{Forward}}\;{\mathrm{primer:}}\;{\mathrm{AGGTCGGTGTGAACGGATTTG;}} \hfill \\ {\mathrm{Reverse}}\;{\mathrm{primer:}}\;{\mathrm{TGTAGACCATGTAGTTGAGGTCA}}{\mathrm{.}} \hfill \\ \end{gathered} \\ \end{array}$$

### Experimental groups

AIH was induced in mice by ConA injection (15 mg/kg, 200ul) via tail vein. The mice were randomly divided into 4 experimental groups (*n* = 6 per group). (1) Naive control group: mice did not undergo any intervention; (2) Untreated group: 30 minu after ConA injection, mice were treated with 200 μl PBS; (3) Unmodified ERC group: thirty minutes after ConA injection, mice were treated with ERCs (1 × 10^6^, suspended in 200 μl PBS); (4) Gal-9 high-expressing ERC group: 30 min after ConA injection, mice were treated with Gal-9 high-expressing ERCs (ERCs transfected with GFP-Gal-9-LVs).

24 h after modeling, all mice were sacrificed and their spleens and parts of liver tissue were ground for flow cytometry. Parts of the collected liver tissues were immersed in 10% formalin solution for pathological staining. The remaining liver tissues and sera were placed in − 80℃ freezer for the following evaluation.

### H&E staining

The liver tissues fixed with 10% formalin were embedded in paraffin and cut into 5 μm sections for H&E staining. Then, the liver injury degree was blindly evaluated by two pathologists based on the liver Knodell scoring system [24].

### Detection of alanine transaminase (ALT) and aspartate transaminase (AST)

The sera levels of ALT and AST were measured by ALT and AST assay kit (Jiancheng Bioengineering Institute Nanjing, China). All the experimental procedures were performed according to the manufacturer instructions and enzyme activities were shown in international unit per liter (IU/L).

### Enzyme-linked immunosorbent assay (ELISA)

Sera and liver tissue homogenates were collected separately for measuring the level of IFN-γ, TNF-α, IL-4, and IL-10. ERCs (with or without GFP-Gal-9-LVs transfected) were also collected to measure Gal-9 protein expressions. The above scheming indicators were all detected by ELISA Kits (DAKEWE, Shenzhen, China) based on the manufacturer's instructions. In order to reduce the error, each sample had two parallel wells.

### Flow cytometry analysis

Fifth-generation ERCs were collected and stained with CD29, CD44, CD45, CD31, and CD90 (ebioscience Inc, USA) fluorescent antibodies, followed by analyzing with the FACS cytometer (BD Biosciences, America). The collected spleens of different groups were ground and made into single-cell suspensions. Then, they were divided into different test tubes and waited for staining. In addition, livers collected from different mouse groups were ground and single cell suspensions were made through filtering a 200-mesh nylon screen. The hepatic lymphocytes were isolated by Percoll gradient separation (top Percoll layer: 40%; bottom Percoll layer: 70%) and divided into different test tubes for staining [25].

Flow cytometry antibodies, including FITC-CD3^+^, Percp-CD8^+^, PE/APC-CD4^+^, PE-CD25^+^, FITC-CD4^+^, APC-FOXP3^+^, Percp-IL17^+^, PE-IFN-γ, APC-IL-4^+^ (ebioscience Inc., San Diego, CA, USA) were used to label CD4^+^ T (CD3^+^CD4^+^), CD8^+^ T (CD3^+^CD8^+^), Th1 (CD4^+^IFN-γ^+^), Th17 (CD4^+^IL-17^+^) and Treg (CD4^+^CD25^+^Foxp3^+^) cell populations. The specific steps were performed as previously described [15]. All the data obtained above were analyzed by using flowjo V10 software.

### Immunohistochemistry staining

The liver tissues fixed in formalin solution were sliced. After deparaffinization, rehydration, antigen retrieval, and endogenous peroxidase inactivation, the slides were collected and incubated with primary rabbit anti-mouse CD4 and CD8 monoclonal antibodies (abcam, Shanghai, China) at 4 °C overnight. Then we developed the signal by using PV9000 kit (Solarbio, Beijing, China). Finally, the slides were counterstained with hematoxylin and observed under a microscope.

### Immunofluorescence staining for in vivo ERC tracking

To track ERCs in vivo, all mice were sacrificed and tissues (livers, spleens, colon, and kidneys) were collected and frozen at − 80 °C. These frozen tissue blocks were cut into 5 μm sections and observed under the fluorescence microscope.

In addition, the frozen liver tissue sections (5 μm) were fixed in acetone for 8 min and then incubated with primary rabbit anti-human Gal-9 antibody (Abcam, USA) at 4 °C overnight. Next, Cy3-goat-anti-rabbit IgG (EΛRTH) was adopted and incubated with the sections at room temperature for 1 h. DAPI was applied to counterstain the nuclei and the slides were photographed under fluorescence microscopy.

### Co-culture of CD4^+^ T cells and ERCs in vitro

Naive CD4^+^ T cells were isolated from C57BL/6 mice splenocytes by CD4^+^ microbeads kit (Precision Biomedicals, Tianjin, China). ERCs with or without GFP-Gal-9-LVs transfection were resuspended and counted as a concentration of 2.5 × 10^4^. CD4^+^ T-cells were activated by stimulating with anti-mouse CD3 (100 ng/ml), CD28 (200 ng/ml) antibodies (ebioscience Inc. USA) and IL-2. Then, we co-cultured the above activated CD4^+^ T cells (5 × 10^5^) with ERCs (2.5 × 10^4^) with a 48-well plate. Inactivated CD4^+^ T cells were used as control. The cell-substrate was harvested on day 6 and its activity was evaluated by a CCK-8 cell counting kit (BOSTER, Wuhan, China) according to the manufacturer's instructions.

### Western blotting

Cell substrates of the co-culture system were washed and lysed in RIPA lysis buffer which contained protease inhibitors. Then, the extracted protein was quantified by the BCA assay kit (Tiangen Biotech, China) and redistributed into the same concentration. The supernatant obtained by lysis was denatured by heating. And the total protein was separated by SDS-PAGE and transferred to PVDF membranes. PVDF membranes were blocked with 5% skim milk and then incubated with primary antibodies: Anti-mouse Lck (1:1000, AF6101, Affinity); Anti-mouse Phospho-Lck (1:1000, AF7194, Affinity); Anti-mouse ZAP70 (1:1000, AF6312, Affinity); Anti-mouse Phospho-ZAP70 (1:1000, AF3312, Affinity); Anti-mouse LAT (1:2000, DF7630, Affinity); Anti-mouse Phospho-LAT (1:1500, AF4380, Affinity) and Anti-mouse Hsp90 (1:1000, abcam) at 4 °C overnight. On the other day, the secondary antibody (Anti-rabbit, 1:2000) was diluted and incubated with the PVDF membranes at room temperature for 30 min. Finally, the strips were developed with ECL solution and photographed with an exposure machine. And the gray values were calculated and analyzed by Image J software.

### Statistical analysis

Data are presented as mean ± SEM. The differences between groups were calculated by using ANOVA analysis (groups ≥ 3) or unpaired t-test (groups = 2). The histograms were made by the prism statistical package (GraphPad Software Inc, USA). *P* values less than 0.05 (*p* < 0.05) was considered statistically significant.

## Results

### Characterization of ERCs

**Fig. 1:**
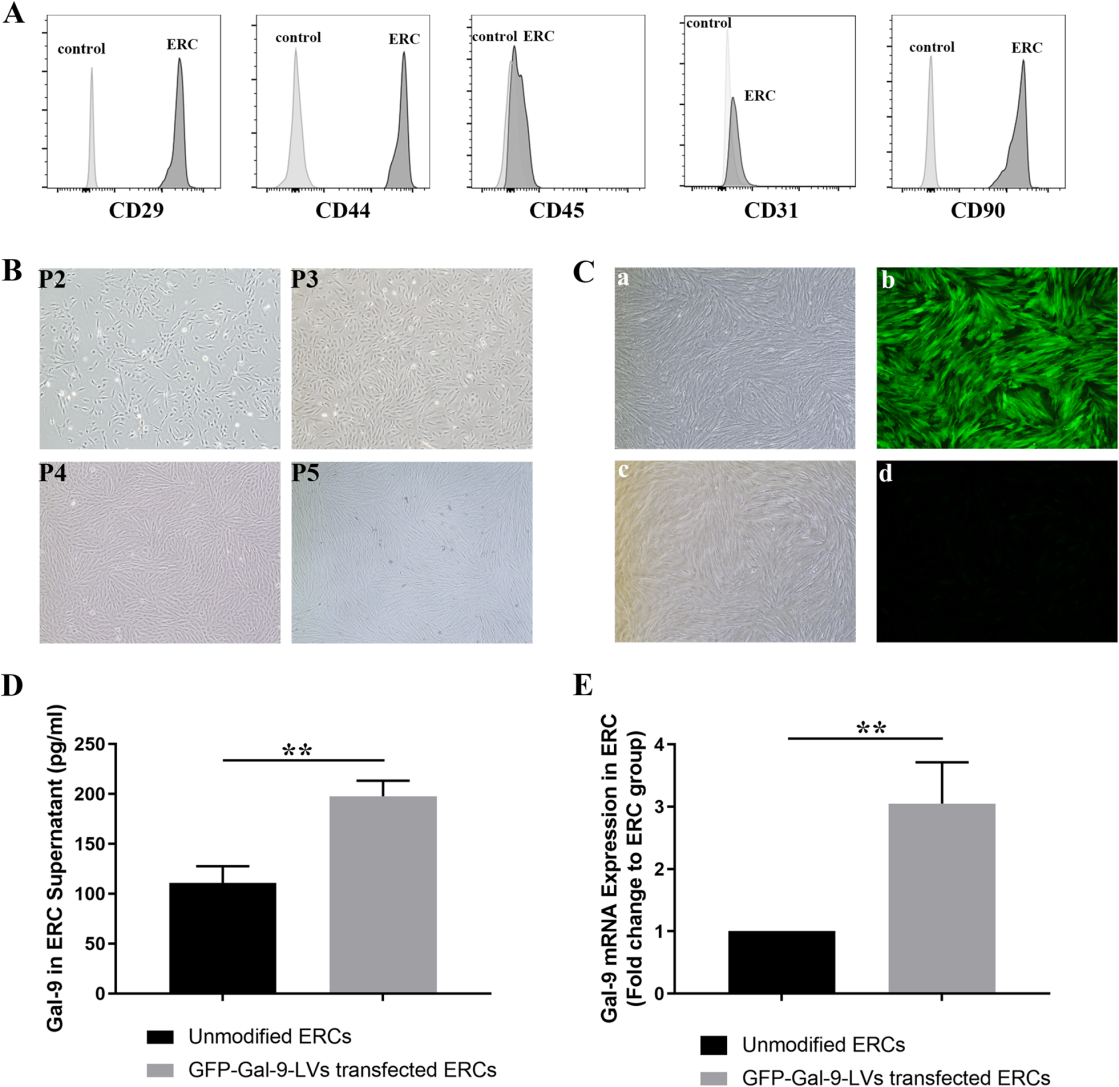
Characterization of ERCs and upregulation of Gal-9 in ERCs. **A** Cell surface markers of ERCs were measured by flow cytometry. ERCs express CD29, CD44, CD90, while lacking of CD45, CD31. **B** Cell morphology images (magnification 40×) of ERCs at P2-5. **C** Brightfield and fluorescence pictures are shown on the left and right side. Representative picture of GFP-Gal-9-LVs transfected ERCs (a-b) and unmodified ERCs(c-d) (magnification 40×). **D** Gal-9 protein expression in unmodified ERCs and GFP-Gal-9-LVs transfected ERCs. n = 3. **p < 0.01. **E** Gal-9 mRNA relative expression in unmodified ERCs and GFP-Gal-9-LVs transfected ERCs. n = 3. **p < 0.01. *p* value was calculated using unpaired t-test. Abbreviations: *ERCs* endometrial regenerative cells, *Gal-9* galectin-9, *P3-5* passage3-5

ERCs, derived from menstrual blood, could be identified by verifying their cell surface markers. Through flow cytometry analysis, we found ERCs highly expressed CD29, CD44, and CD90, but did not express CD45 and CD31 (Fig. 1A). Additionally, we observed that these ERCs exhibited a spindle shape and grew in radial clusters in 2nd–5th generations (Fig. 1B).

### Gal-9 is significantly upregulated in GFP-Gal-9-LVs transfected ERCs

In order to clarify the transfection status, we observed the transfected ERCs with a fluorescence microscope. The fluorescence pictures of GFP-Gal-9-LVs transfected ERCs and unmodified ERCs were shown in Fig. 1C-b, C-d, respectively. There is a significant higher expression of green fluoresce protein in GFP-Gal-9-LVs transfected ERCs (Fig. 1C-b), but not in unmodified ERCs (Fig. 1C-d), indicating that GFP-Gal-9-LVs transfected ERCs with a higher expression of Gal-9. Then, we further analyzed Gal-9 expression in the cell lysates of unmodified ERCs and GFP-Gal-9-LVs transfected ERCs by ELISA. We found that Gal-9 expression in GFP-Gal-9-LVs transfected ERCs was significantly higher than that of unmodified ERC group (*p* < 0.01, Fig. 1D). The same result was proved by RT-PCR at transcript level (*p* < 0.01, Fig. 1E). We also found that transfection of negative control lentivirus vector had no effect on the expression of Gal-9 in ERCs (*Data not shown*). Taken together, these results demonstrated that Gal-9 was significantly upregulated in ERCs with GFP-Gal-9-LVs transfection and this finding could provide a basis for the following in vitro and in vivo studies.

### Gal-9 mediates ERCs to alleviate ConA-induced experimental AIH in mice

**Fig. 2:**
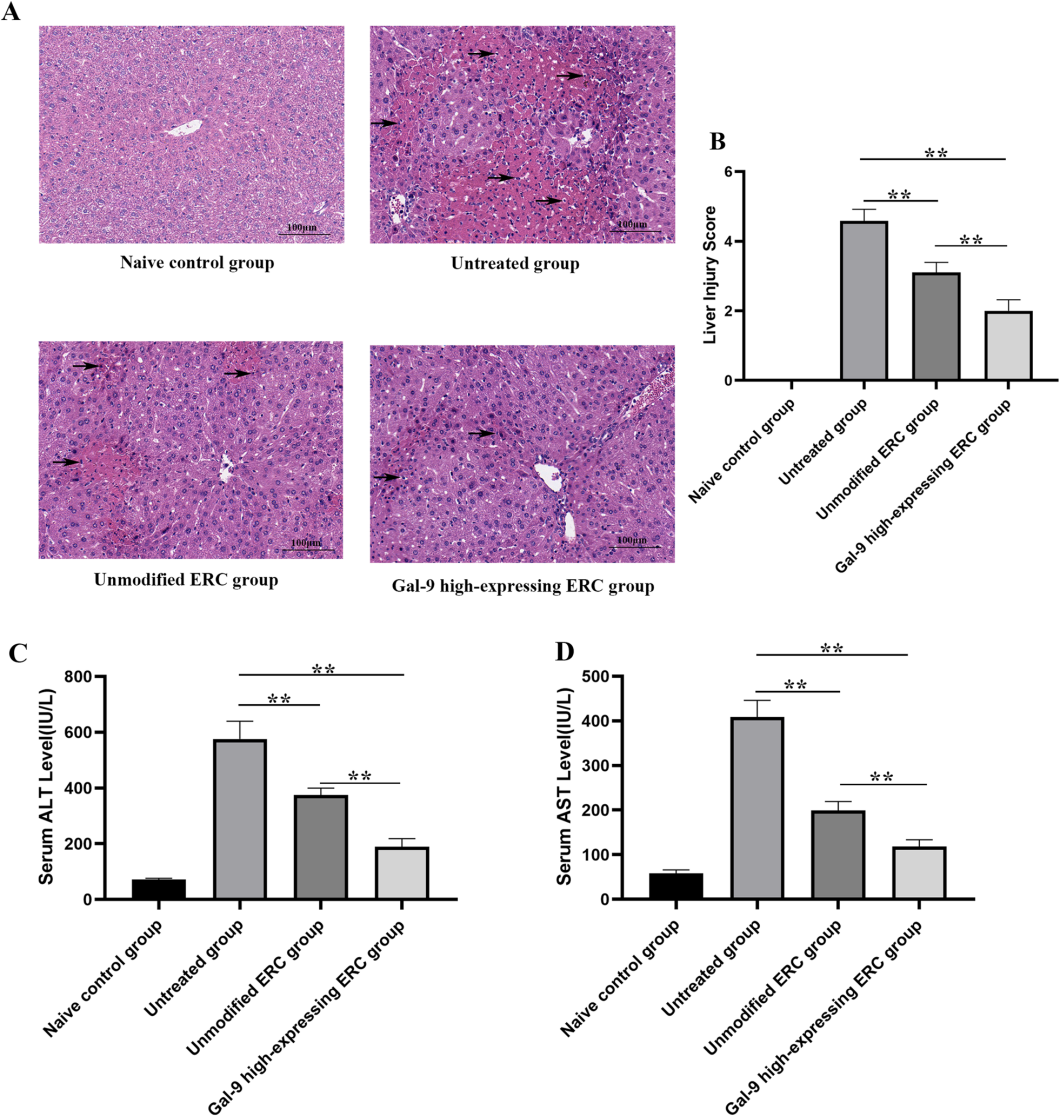
Gal-9 mediates ERCs to alleviate ConA-induced experimental AIH in mice. **A** Representative images of liver tissues in each group (H&E staining), Black arrows indicate inflammation cell infiltration. **B** Knodell score was applied to quantified liver damage. **C**, **D** Serum ALT and AST levels. The differences between groups were calculated by using ANOVA. *p < 0.05, **p < 0.01. Abbreviations: *H&E* hematoxylin and eosin, *ALT* alanine transaminase, *AST* aspartate transaminase, *ANOVA* one-way analysis of variance

To examine the potential therapeutic effect of Gal-9 high-expressing ERCs on ConA-induced experimental AIH, the conditions of liver tissue in different groups were determined by HE staining. As shown in Fig. 2A, pathology of the untreated group showed with clusters of inflammatory cell infiltration in the parenchyma and portal area, as well as punctate necrosis, focal necrosis, and extensive necrosis. The liver damage was significantly alleviated with the treatment of Gal-9 high-expressing ERCs.

Moreover, liver damage degree was also quantified by the liver Knodell score (Fig. 2B). In line with the pathological manifestations, the unmodified ERC group had less damage in the liver, when compared with that of the untreated group (*p* < 0.01). The liver damage was significantly attenuated in the Gal-9 high-expressing ERC group (unmodified ERC group vs. Gal-9 high-expressing ERC group, *p* < 0.01).

The serum levels of ALT and AST were also tested to assess liver function. ALT and AST in the unmodified ERC treated group were both significantly lower than that of the untreated group (Fig. 2C, D, unmodified ERC group vs. untreated group, *p* < 0.01), while the levels of ALT and AST in the Gal-9 high-expressing ERC group were lower than those of unmodified ERC group (*p* < 0.01). Given above, these results suggested that ERCs could alleviate AIH and restore liver function, and this therapeutic effect was markedly enhanced by Gal-9 high expression in ERCs.

### Gal-9 elevated the immunoregulatory capacity of ERCs in inhibiting the proliferation of CD4^+^ and CD8^+^ T cells in AIH mice

**Fig. 3:**
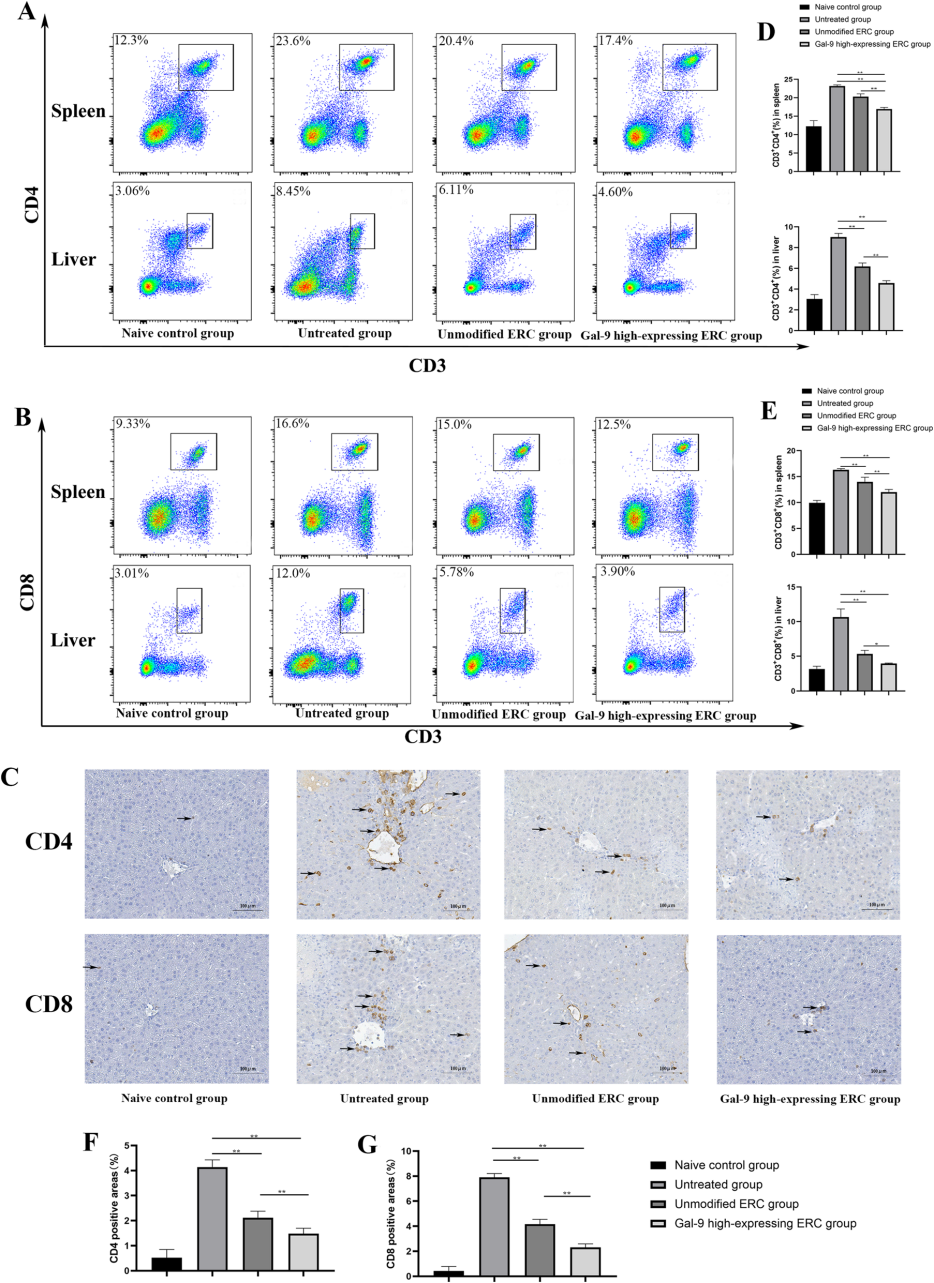
Gal-9 elevates the immunoregulatory capacity of ERCs in inhibiting the proliferation of CD4^+^ and CD8^+^ T cells in AIH mice. 24 h after model induction, single-cell suspensions were made from spleens of different groups. Splenocytes and hepatocytes were stained with anti-mouse CD3 FITC, anti-mouse CD4 PE, anti-mouse CD8 Percp antibodies, and then analyzed by flow cytometry. **A** Representative dot plots depict the percentage of CD4^+^ T cell (CD3^+^CD4^+^). **B** Representative dot plots depict the percentage of CD8^+^ T cell (CD3^+^CD8^+^). **C** Immunohistochemical staining of CD4^+^ and CD8^+^ T cell infiltration in the liver. The black arrows show the positive staining areas. **D**, **E** Percentage of CD4^+^ T cells and CD8^+^ T cells in the spleen and liver. **F**, **G** CD4^+^ T and CD8^+^ T cells were quantified by using ImageJ software to identify and calculate the brown-black positive areas. The differences between groups were calculated using ANOVA. *p < 0.05, **p < 0.01

T cells are the cardinal cells involved in the pathogenesis of AIH [1, 26]. In order to verify whether Gal-9 high expression plays an important role in modulating T cell activation in this AIH model, splenocytes and Hepatocytes from different groups were harvested and detected by flow cytometry. Representative dot plots of CD3^+^CD4^+^ and CD3^+^CD8^+^ T cells of spleen and liver were displayed in Fig. 3A, B, and the quantitative analysis of CD3^+^CD4^+^ and CD3^+^CD8^+^ T cell of spleen and liver populations were shown in Fig. 3D, E. The population of CD4^+^ T or CD8^+^ T cells in the unmodified ERC group was significantly lower than that in the untreated group (spleen: CD4^+^ T cells: untreated group vs. unmodified ERC group, *p* < 0.01; CD8^+^ T cells: untreated group *vs.* unmodified ERC group, *p* < 0.01; liver: CD4^+^ T cells: untreated group *vs.* unmodified ERC group, *p* < 0.01; CD8^+^ T cells: untreated group vs. unmodified ERC group, *p* < 0.01). These T cell populations were further reduced in the Gal-9 high-expressing ERC group (spleen: CD4^+^ T cells: unmodified ERC group vs. Gal-9 high-expressing ERC group, *p* < 0.01; CD8^+^ T cells: unmodified ERC group vs. Gal-9 high-expressing ERC group, *p* < 0.01; liver: CD4^+^ T cells: unmodified ERC group vs. Gal-9 high-expressing ERC group, *p* < 0.01; CD8^+^ T cells: unmodified ERC group vs. Gal-9 high-expressing ERC group, *p* < 0.05).

To observe the immune infiltration of CD4^+^ T and CD8^+^ T cells more intuitively in locally target organs, we have stained CD4^+^ T and CD8^+^ T cells in the liver by immunohistochemical staining (Fig. 3C). For quantitative analysis, the brown-black positive areas representing CD4^+^ cells or CD8^+^ cells were identified and calculated by ImageJ software (Fig. 3F, G). The numbers of both of CD4^+^ and CD8^+^ cell infiltrations were significantly reduced in the Gal-9 high-expressing ERC group. The trend of cell populations was consistent with the results observed in spleens.

Collectively, these data suggest that Gal-9 elevates the immunoregulatory capacity of ERCs in downregulating the populations of CD4^+^ and CD8^+^ T cells in this AIH model.

### Gal-9 mediates ERCs to reduce Th1 and Th17 cell differentiation, but improve Treg population in vivo

**Fig. 4:**
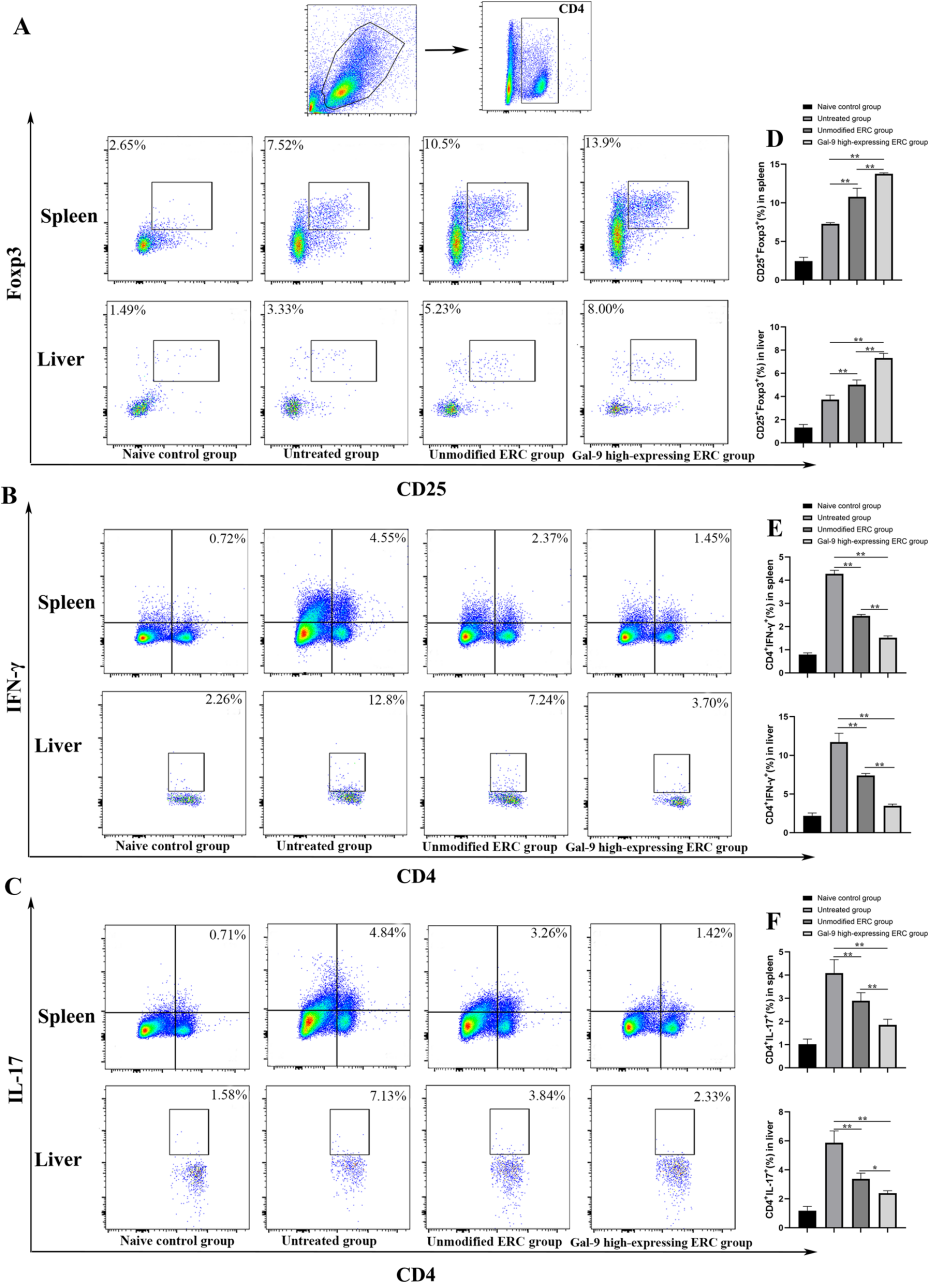
Gal-9 mediates ERCs to reduce Th1 and Th17 cell differentiation, but improve Treg population in vivo*.* Single-cell suspensions were obtained from the spleen and liver. Cells were stained with anti-mouse CD4, IFN-γ, IL-17, CD25, Foxp3 antibodies. **A** Representative dot plots depict the percentage of Tregs (CD4^+^CD25^+^Foxp3^+^) in the spleen and liver. **B** Representative dot plots depict the percentage of Th1 (CD4^+^IFN-γ^+^) cells in the spleen and liver. **C** Representative dot plots depict the percentage of Th17 (CD4^+^IL-17^+^) cells in the spleen and liver. **D** Percentage of Tregs in spleen and liver. **E** Percentage of Th1 cells in spleen and liver. **F** Percentage of Th17 cells in spleen and liver. The differences between groups were calculated using ANOVA. *p < 0.05, **p < 0.01. Abbreviations: *Treg* regulatory T cells, *ANOVA* one-way analysis of variance

This experiment has proved Gal-9 high-expressing ERCs play a role in modulating CD4^+^ T cell proportions. To further clarify their role in regulating the subtype of CD4^+^ T cells, the proportions of Th1, Th17, and Treg cells in the mouse spleen and liver were measured by flow cytometry. As shown in Fig. 4B, E, the percentage of Th1 cells was lower in the unmodified ERC group, when compared with that of the untreated group (spleen, *p* < 0.01; liver, *p* < 0.01). Moreover, the Th1 cell percentage was further reduced in the Gal-9 high-expressing ERC group (spleen: unmodified ERC group *vs.* Gal-9 high-expressing ERC group, *p* < 0.01; liver: unmodified ERC group *vs.* Gal-9 high-expressing ERC group, *p* < 0.01). Consistently, the same trend was found in Th17 cells (Fig. 4C, F, spleen: untreated group *vs*. unmodified ERC group, *p* < 0.01; unmodified ERC group *vs*. Gal-9 high-expressing ERC group, *p* < 0.01, liver: untreated group *vs*. unmodified ERC group, *p* < 0.01; unmodified ERC group vs. Gal-9 high-expressing ERC group, *p* < 0.05).

Tregs were generally recognized as the immunosuppressive CD4^+^ T cells, and have been reported to a role in impeding the progression of AIH [27]. In the present study, we also measured Treg proportion in splenocytes. The percentage of Tregs was the highest in the Gal-9 high-expressing ERC group (Fig. 4A, D, spleen: unmodified ERC group *vs*. Gal-9 high-expressing ERC group, *p* < 0.01, liver: unmodified ERC group *vs*. Gal-9 high-expressing ERC group, *p* < 0.01), indicating that Gal-9 high-expressing ERCs have an effect in promoting Treg differentiation.

Collectively, these results showed that Gal-9 high-expressing ERCs could mitigate the systematic and local immune response in AIH mice by reducing the proportions of Th1 and Th17 cells and increasing the proportion of Tregs in the liver and spleen.

### Gal-9 mediates ERCs to reduce the inflammatory response in AIH mice

**Fig. 5:**
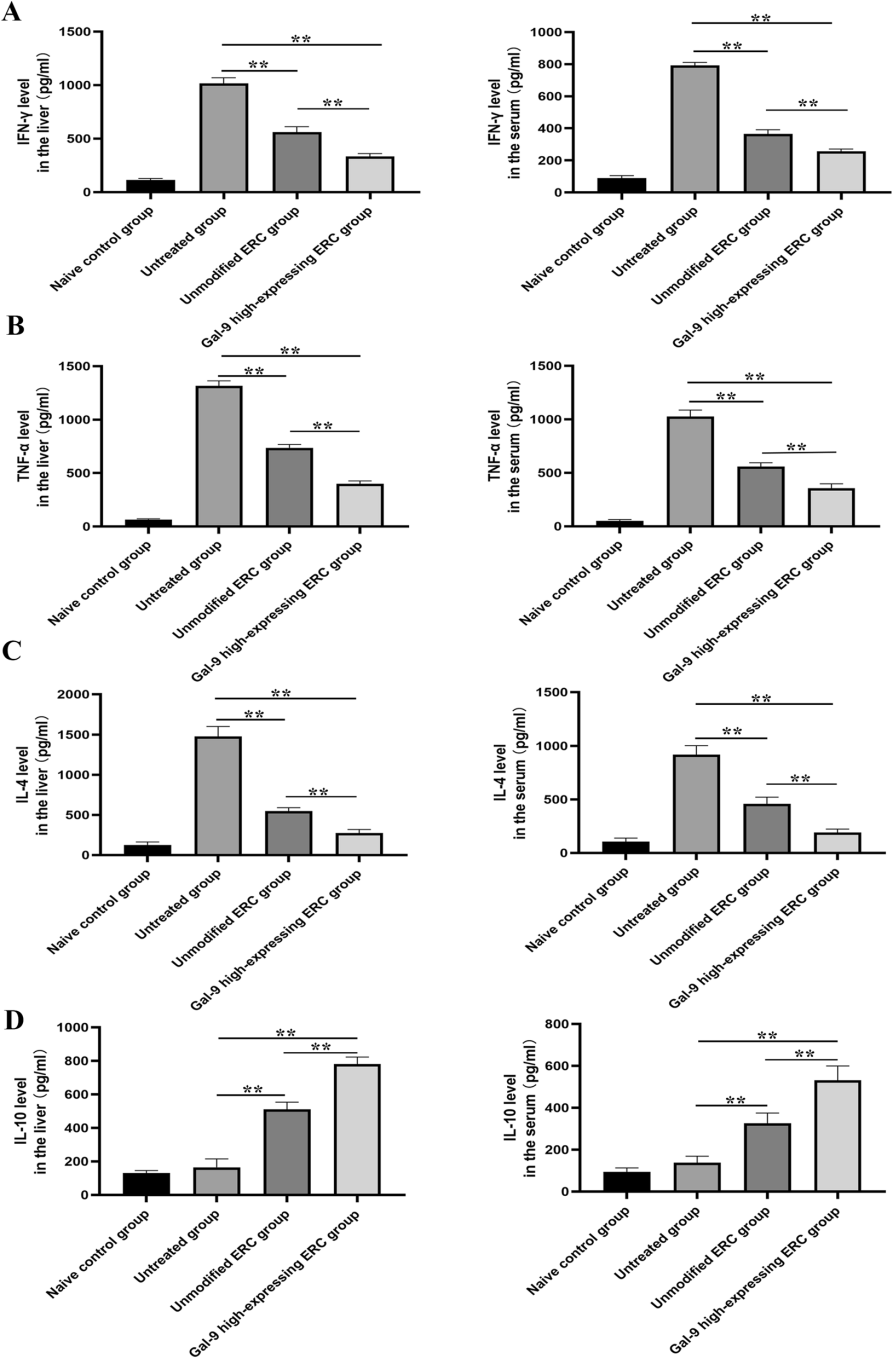
Gal-9 mediates ERCs to reduce the inflammatory response in AIH mice. Levels of IFN-γ (**A**), TNF-α (**B**), IL-4 (**C**), IL-10 (**D**) in sera and livers were detected by ELISA. The differences between groups were calculated using ANOVA. *p < 0.05, **p < 0.01. Abbreviations: *ELISA* enzyme-linked immunosorbent assay

To analyze cytokine profiles in AIH mice, we have measured the levels of IFN-γ, TNF-α, IL-4, and IL-10 in both the sera and liver homogenate from different groups. As shown in Fig. 5, the levels of pro-inflammatory cytokines (IFN-γ, TNF-α, and IL-4) were significantly reduced in the sera and liver homogenate after treatment with unmodified ERCs (*vs.* Untreated group, *p* < 0.01). Furthermore, these cytokine levels further declined after the infusion with the Gal-9 high-expressing ERCs (unmodified ERC group vs. Gal-9 high-expressing ERC group, liver: IFN-γ, *p* < 0.01; TNF-α, *p* < 0.01, IL-4, *p* < 0.01; serum: IFN-γ, *p* < 0.01; TNF-α, *p* < 0.01, IL-4, *p* < 0.01). In addition, the level of anti-inflammatory factors (IL-10) was notably elevated after the treatment with unmodified ERCs (vs. untreated group, *p* < 0.01), and they tend to be increased when treated with Gal-9 high-expressing ERCs (unmodified ERC group vs. Gal-9 high-expressing ERC group, liver *p* < 0.01, serum *p* < 0.01). Given together, it suggested that Gal-9 high-expressing ERCs actively participated in regulating cytokine profile during the process of inflammation in this AIH model.

### Tracking transplanted ERCs in vivo

**Fig. 6:**
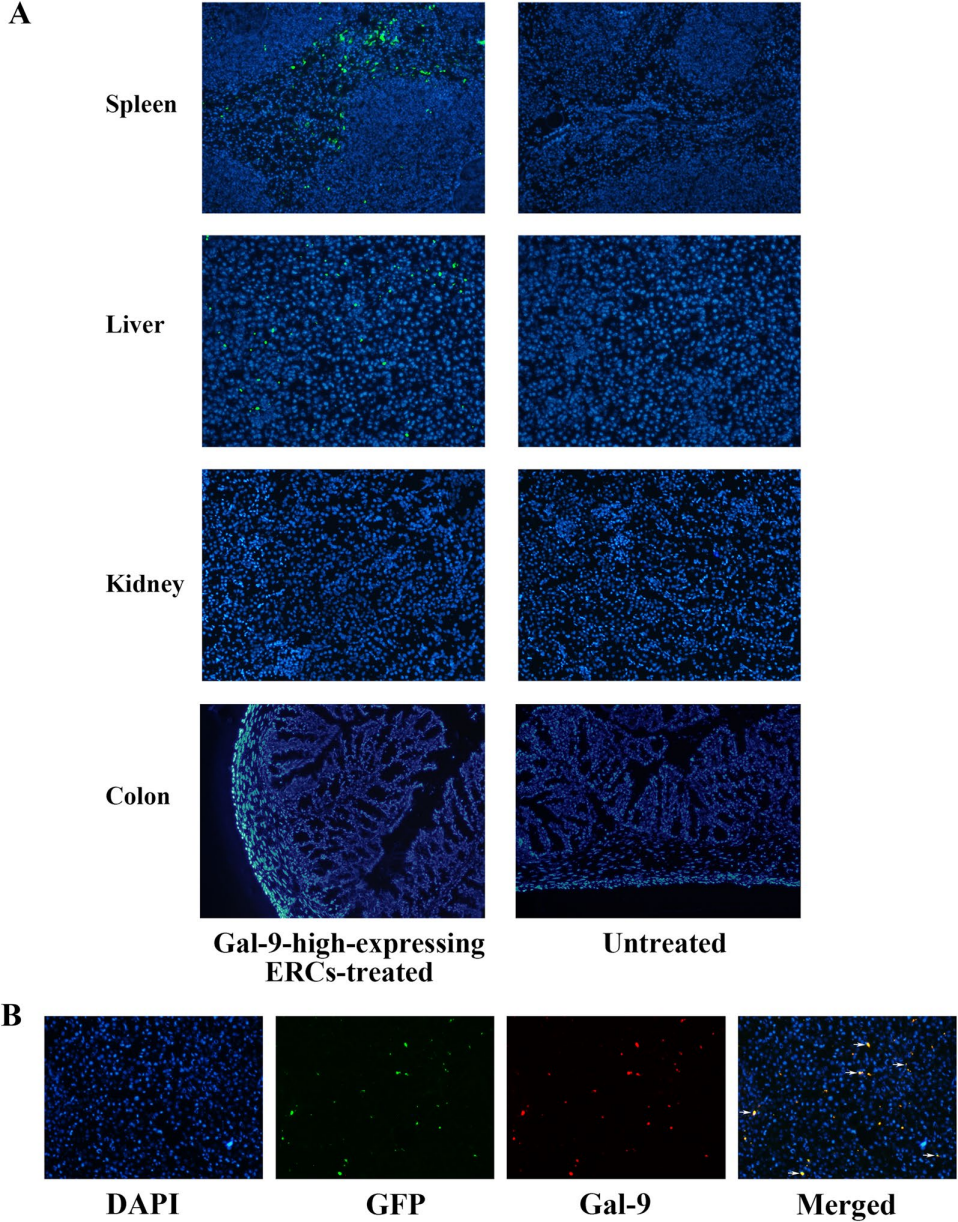
Tracking transplanted ERCs in vivo. **A** In vivo tracking shows that Gal-9-high-expressing ERCs (green staining) were detected in liver and spleen but not in kidney and colon (magnification 100×). **B** Immunofluorescence showing the co-localization of ERC (green staining) and Gal-9 (red staining) in liver. (magnification 100×). Abbreviations: *GFP* green fluorescent protein

MSCs have been demonstrated to migrate and accumulate in the damaged organs by chemotaxis factors [28]. To study whether Gal-9 high-expressing ERCs can migrate to the damaged liver tissues in this AIH model, we have injected ERCs into the mice through the tail vein after the AIH induction. 24 h later, liver, kidney, colon, and spleen were harvested, proceeded, and photographed under fluorescence microscope. As shown in Fig. 6A, Gal-9 high-expressing ERCs with GFP (green staining) were detected in the liver (injured organ) and spleen (lymphatic organ), but not found in the kidney (naive organ) and colon (naive organ). For comparison, we did not find any green fluorescent protein in the untreated mice. Moreover, in order to observe the Gal-9 protein more intuitively, we stained the above collected liver tissues with Gal-9 primary antibody and Cy3t-anti-IgG secondary antibody. As shown in Fig. 6B, we could observe that ERCs, accumulating in the liver tissue, highly expressed Gal-9 (red staining). Through the above results, we confirmed that ERCs could migrate to the damaged liver, and speculated that ERCs might play a protective role in this AIH model through Gal-9 high-expression.

### Inhibition of T cell proliferation by Gal-9 high-expressing ERCs is associated with Lck/ZAP70/LAT pathway

**Fig. 7:**
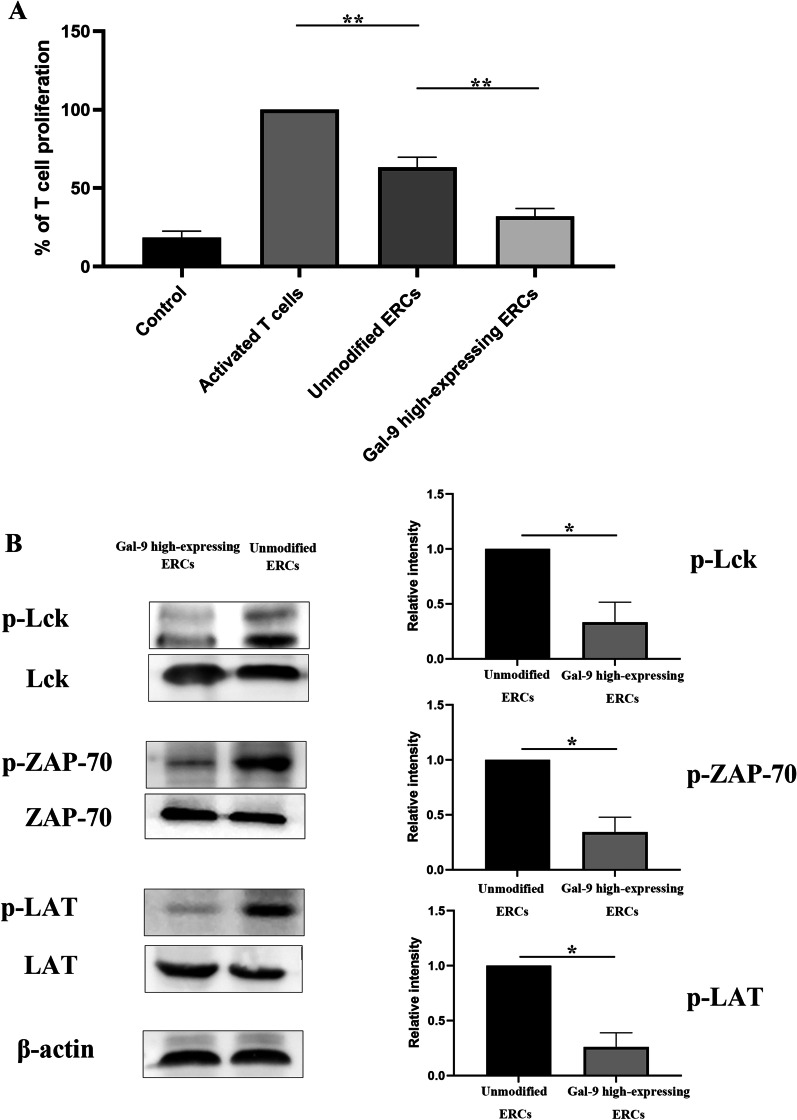
Inhibition of T cell proliferation by Gal-9 high-expressing ERCs is associated with Lck/ZAP70/LAT pathway. The naïve CD4^+^ T cells in the mouse spleen were collected by magnetic bead, and then the harvested CD4^+^ T cells were co-cultured with unmodified ERCs or Gal-9 high-expressing ERCs for 6 days. **A** CD4^+^ T cell proliferation was determined by CCK-8 assay. **B** The protein expressions and phosphorylation form protein expressions of Lck, ZAP-70, LAT were detected by western blot. The differences between groups were calculated by using ANOVA. *p < 0.05, **p < 0.01. Abbreviations: *Lck* lymphocyte-specific protein tyrosine kinase, *ZAP-70* zeta-chain-associated protein kinase 70, *LAT* linker for activation of T cells

We have found that Gal-9 high-expressing ERCs could modulate CD4^+^ T cell proliferation in splenocytes and inhibit CD4^+^ cell infiltration in damaged livers (Fig. 3). In order to further evaluate the modulation effect of Gal-9 high-expressing ERCs, we co-cultured Gal-9 high-expressing ERCs with activated CD4^+^ T cells in vitro. As shown in Fig. 7A, through CCK-8 test, we found that Gal-9 high-expressing ERCs had a significant effect in inhibiting the proliferation of CD4^+^ T cells, when compared with unmodified ERCs (*p* < 0.01).

In order to elucidate the possible pathway of Gal-9 protein acting on CD4^+^ T cells, we measured phosphorylated Lck, ZAP-70, and LAT protein expressions in CD4^+^ T cells, which was related to T cell proliferation. As shown in Fig. 7B, the results showed that phosphorylated Lck, ZAP-70, and LAT proteins in activated CD4^+^ T cells were significantly decreased after co-culturing with Gal-9 high-expressing ERCs (vs. unmodified ERC group, *p* < 0.05). Given above, the process that Gal-9 protein mediates ERCs to inhibit T cell proliferation is likely through downregulation of the Lck/ZAP70/LAT pathway.

## Discussion

**Fig. 8:**
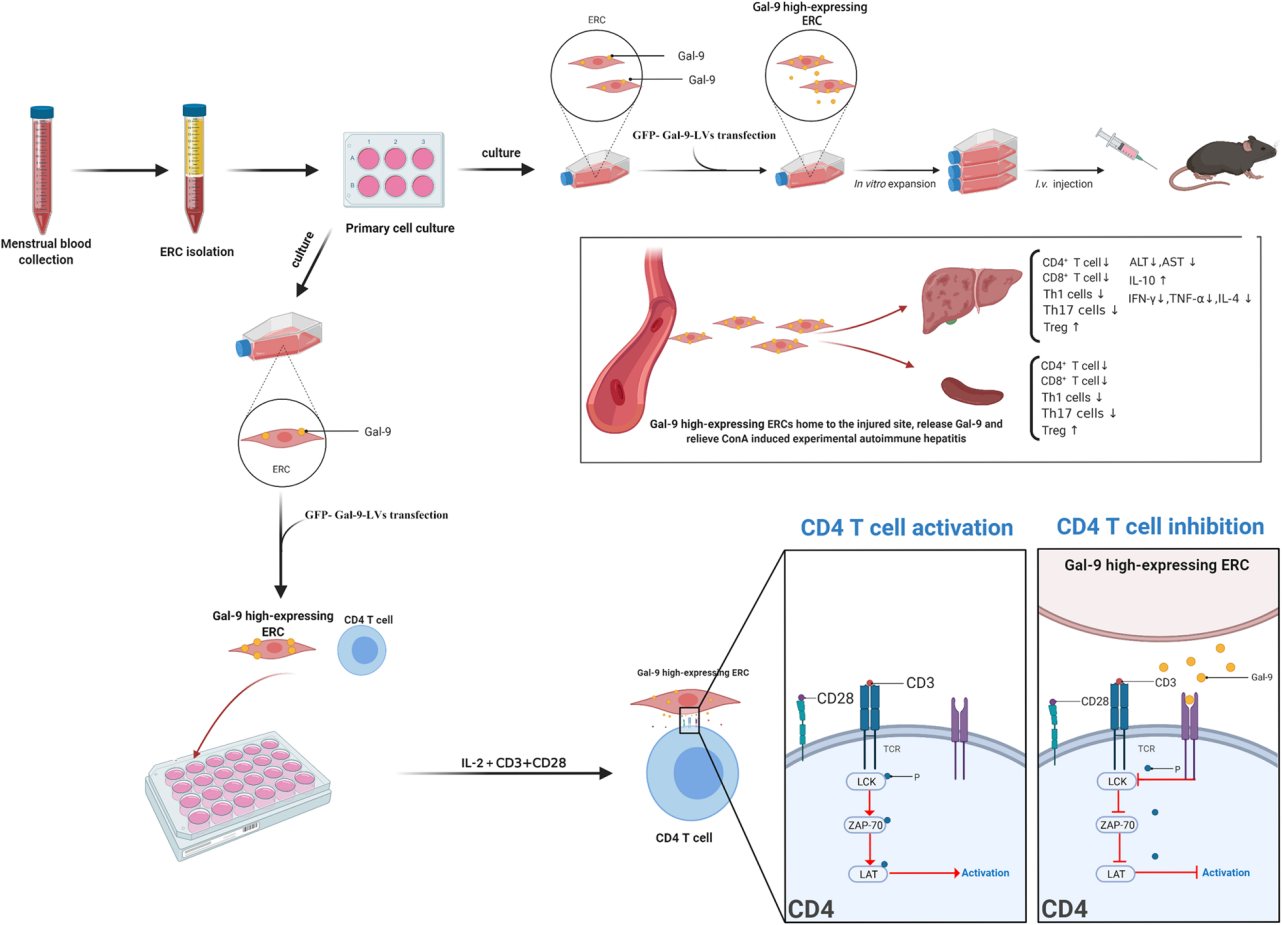
Application of Gal-9 high-expressing ERCs in ConA-induced experimental autoimmune hepatitis in mice. ERCs are isolated from menstrual blood and transfected with GFP-Gal-9-LVs in vitro. After expansion, in vitro co-culture and in vivo administration, the results show that Gal-9 high-expressing ERCs effectively restore the liver function, ameliorate liver pathological damage, inhibit CD4^+^ and CD8^+^ T cell proliferation and suppress Th1 and Th17 cell response in the hepatitis mice. Additionally, Gal-9 high-expressing ERCs also markedly enhance the level of IL-10, but reduce the levels of IFN-γ, TNF-α, and IL-4 in mouse sera and liver. In addition, it also indicates that Gal-9 high-expressing ERCs inhibit CD4^+^ T cell activation in vitro, and proves that this inhibition is associated with the inactivation of Lck/ZAP-70/LAT pathway. These results would provide a novel idea for the clinical treatment of AIH. (Created by using www.biorender.com software)

In this study, we have transfected ERCs with GFP-Gal-9-LVs and found that Gal-9 expression in ERCs increased significantly with the GFP-Gal-9-LV transfection. And we have proved that Gal-9 high-expressing ERCs can further reduce the progression of ConA-induced experimental autoimmune hepatitis, by reducing the infiltration of CD4^+^ and CD8^+^ T cells in the liver, suppressing Th1 and Th17 cell response, inhibiting CD4^+^ and CD8^+^ T cell proliferation in the hepatitis mice, and regulating inflammatory factors. In addition, we have also proved that Gal-9 high-expressing ERCs inhibited CD4^+^ T cell activation, which was trough regulating the LCK/ZAP-70/LAT pathway (Fig. 8).

The ERCs were harvested from volunteer’s menstrual blood by using menstrual cup, and the initial collected cell number was about 10^8^. After 24 h culture, the adherent cell number was about 5 × 10^5^ per well. Additionally, we observed that these cells exhibited a spindle shape and grew in radial clusters. We also verified the surface markers of ERCs which highly express some MSC markers such as CD29, CD44, CD90. In addition, the absence of expressions of CD45 and CD31 on ERCs excludes the origin of hematopoeitic cells and endothelial cells, respectively. These experimental results suggesting that the collected ERCs were purified and homogeneous.

Specifically, we harvested Gal-9 high-expressing ERCs by GFP-Gal-9-LV transfection, and constructed AIH model by ConA injection. It is worth noting that male mice were selected as animal models in this study to keep the stable and uniform of the experimental results. Then, 30 min after the modeling, unmodified ERCs and Gal-9 high-expressing ERCs were administrated into the AIH mice separately (*i.v.*). We observed that ERCs could migrate to the liver (green staining) and spleen (green staining), but not detected in the colon and kidney. In addition, through the immunofluorescent co-localization staining, we found that ERCs (green staining) co-located with Gal-9 protein (red staining) in the AIH mouse liver, indicating that ERCs with Gal-9 high expressing could migrate to the damaged organs. Studies have shown that ERCs can home to damaged areas and survive in body for 14 days [29, 30]. From this, we infer that the persistence of Gal-9 high-expressing ERCs in injured organs would inevitably produce a large amount of Gal-9 protein, which would promote the repair of the injury.

In addition, we have also evaluated serum levels of ALT and AST in different groups. ALT and AST are mainly secreted by liver cells. When liver cells are damaged, ALT and AST will be released into the blood in large quantities [31]. In this study, both ALT and AST levels were significantly surged after induction of AIH, but were markedly decreased with the treatment of unmodified ERCs. Additionally, their levels were further reduced by Gal-9 high-expressing ERC therapy. This is consistent with our pathological staining findings. In ConA-induced AIH, the main manifestations are inflammatory cell infiltration, massive necrosis of hepatocytes, vascular congestion and dilation, and hepatic sinusoidal structural disorders [32]. Knodell score is used to quantify the degree of liver damage, which is one of the most effective indicators for evaluating early liver damage. It is shown that livers in Gal-9 high-expressing ERC group have the lowest damage score with the mildest pathological changes. This result suggests that Gal-9 mediates the therapeutic effects of ERCs in attenuation of AIH.

CD4^+^ T cells were reported to play a critical role in ConA-induced hepatitis in wild-type mice. Through transferring CD8^+^ T cell into Rag2^−/−^ mice, perforin-based CD8^+^ T cell was found to initiate IL33/ILC2 axis to exacerbate the liver injury in ConA-induced hepatitis [33]. In this study, we have also detected CD4^+^ and CD8^+^ T cell proportions in the mouse splenocyte. It has been found that the numbers of both CD4^+^ and CD8^+^ T cells were reduced in the unmodified ERC treated group, and further decreased in the Gal-9 high-expressing ERC group, indicating the immunomodulatory effect of Gal-9-mediated ERCs in attenuation of experimental AIH.

Considering the local T cell variation, we have further analyzed intra-liver CD4^+^ and CD8^+^ T cell infiltration by immunohistochemical staining. In addition, population of CD4^+^ and CD8^+^ T cells in the liver were detected by flow cytometry. Coincidentally, the data obtained from immunohistochemistry and flow cytometry proved that Gal-9 high-expressing ERCs can further reduce the infiltration and populations of CD4^+^ and CD8^+^ T cells in the liver.

In order to further evaluate the influence of the differentiation and proportion changes in the CD4^+^ T cell subsets on the development of experimental AIH, we have detected CD4^+^ Th1 and Th17 cell proportions in mouse splenocytes and hepatocytes. The results showed that Gal-9 high-expressing ERCs could significantly inhibit the proliferation of Th1 and Th17 cells. By analyzing these results, we speculated that the modulation effect of ERCs was mainly based on Gal-9.

CD4^+^Foxp3^+^ Tregs are considered to be important regulatory cells for maintaining immune tolerance [34, 35]. In our previous research, we found that ERCs effectively enhanced Treg population [36]. Similarly, in this present study, the proportion of Tregs was significantly increased by the treatment with ERCs. Interestingly, Treg proportion was further increased when the mice were treated with ERCs with enhanced expression of Gal-9, accompanied by markedly ameliorated AIH. Thus, we speculate that Gal-9 may play an important role in attenuation of AIH. It has been documented that recombinant Gal-9 protein could promote the conversion of CD4^+^ T cells into Foxp3^+^ Tregs in a dose-dependent manner [29]. Our current study has further demonstrated that ERCs with Gal-9 expression participated in the induction of Tregs, and this effect of Treg induction was strengthened by ERCs with much increased expression of Gal-9. Given together, it is rational to believe that Gal-9 mediates ERCs in promoting Treg induction.

A variety of cytokines play an important role in autoimmune hepatitis [30, 37, 38]. IFN-γ is a cytokine mainly produced by T cells and could initiate a cytotoxic response to hepatocytes in AIH model [37]. TNF-α is mainly produced by activated monocytes/macrophages and participates in the pathological damage of multiple autoimmune diseases. Pretreatment of mice with anti-mouse TNF-α antiserum can protect them from ConA-induced hepatitis [30]. IL-4 can upregulation eotaxin in liver cells, thereby recruiting eosinophils and neutrophils to the liver [39, 40]. In the present study, we have tested the above three cytokine expressions, and the results showed that IFN-γ, TNF-α, and IL-4 expressions in the liver and the sera were both reduced by ERC treatment. Through comparison, it has been found that AIH mice treated with Gal-9 high-expressing ERCs have lower levels of inflammatory factors.

In this study, we have also tested the anti-inflammatory factor IL-10. The results showed that the level of IL-10 was significantly increased by unmodified ERCs, and was further upregulated by Gal-9 high-expressing ERCs, leading to much reduced severity of hepatitis. In summary, Gal-9 can enhance the therapeutic effect of ERCs on attenuation of AIH by reducing the levels of pro-inflammatory cytokines, and enhancing the level of the anti-inflammatory factor.

In the current study, we observed that ERC administration could affecting the proliferation of CD4^+^ T cells, but its mechanism is not fully understood. Therefore, we carried out the in vitro co-culture experiment (Gal-9 high expressing ERCs and activated CD4^+^ T cells) to further clarify the role of Gal-9 in ERCs. The CCK-8 test results showed that the therapeutic effect of Gal-9 high expressing ERCs was more potent than that of unmodified ERCs in inhibition of CD4^+^ T cell activation and proliferation. Thus, we speculate that Gal-9, expressed in ERCs, plays an important role in the regulation of CD4^+^ T cells. Additionally, we evaluated several proteins in the above in vitro collected CD4^+^ T cells, which are related to T cell activation [41]. The results showed that the phosphorylated proteins Lck, ZAP-70, and LAT increased significantly after co-culturing with Gal-9 high expressing ERCs. Therefore, it is speculated that Gal-9 expressed in ERCs participated in T cell regulation, which is associated with downregulation of the Lck/ZAP70/LAT pathway.

In this study, we have proved the effect of Gal-9 in enhancing the therapeutic effect of ERCs in a mouse model of ConA-induced AIH. Our results are encouraging, but the in-depth mechanism studies are warranted in different hepatitis models [42].

## Conclusions

This study has verified the therapeutic effect of Gal-9-mediated ERCs in attenuation of ConA-induced AIH. The results have demonstrated that Gal-9 high-expressing ERCs restored liver function, ameliorated liver pathological damage, decreased the number of CD4^+^ and CD8^+^ cell, and inhibited Th1 and Th17 cell proliferation in the hepatitis mice. Cell tracking has also confirmed that ERCs can migrate to the damaged livers and the lymphoid organ (spleens). Furthermore, the in vitro study has indicated that the inhibition of CD4^+^ T cell proliferation by Gal-9-mediated ERCs was associated with the down-regulation of p-Lck/p-ZAP70/p-LAT pathway. Overall, this study would form a basis and provide a novel perspective for the clinical treatment of AIH.

## Data Availability

All data generated or analyzed during this study are included in this article.

## Abbreviations

AIH: Autoimmune hepatitis
ERCs: Endometrial regenerative cells
Gal-9: Galectin-9
MSCs: Mesenchymal stromal cells
TIM-3: T-cell immunoglobulin and mucin-domain containing-3
Tregs: T regulatory cells
GFP-Gal-9-LVs: Lentivirus vector carrying LGALS9 gene and encoding green fluoresce protein
RT-PCR: Real-time polymerase chain reaction
ALT: Alanine transaminase
AST: Aspartate transaminase
ELISA: Enzyme-linked immunosorbent assay
